# Supplementary material for: Study on collateral sensitivity of tigecycline to colistin-resistant Enterobacter cloacae complex
Source: Microbiol Spectr. 2025 May 23;13(7):e03310-24. doi: 10.1128/spectrum.03310-24 (PMC12210861; doi:10.1128/spectrum.03310-24)
Supplement: Supplemental tables — Table S1, clinical isolates of carbapenem-resistant Enterobacter cloacae complex (CRECC) with colistin resistance; Table S2, primers used in this study. [file spectrum.03310-24-s0001.docx]

Table S1. Clinical isolates of carbapenem-resistant *Enterobacter cloacae* complex (CRECC) with colistin resistance.

| Isolates | Source | Date | NCBI accession |
| --- | --- | --- | --- |
| CRECC401 | Bronchoalveolar lavage fluid | 30/5/2021 | JAMYDI000000000 |
| CRECC402 | Sputum | 24/9/2020 | JAMYDH000000000 |
| CRECC405 | Bronchoalveolar lavage fluid | 27/3/2021 | CP091492-CP091495 |
| CRECC414 | Sputum | 13/10/2021 | CP091481-CP091485 |
| CRECC416 | Urine | 22/12/2021 | CP133351-CP133355 |

Table S2. Primers used in this study.

(1) **Used for *Enterobacter asburiae* and *Enterobacter cloacae.***

| Primers | Sequence (5' → 3') |
| --- | --- |
| **Primers for qRT-PCR** | |
| Q-*phoQ*-F | GTGGCGGCGTGGTGGAG |
| Q-*phoQ*-R | TCAGCAGGCGGTTGAGGTTAC |
| Q-*arnA*-F | AGGTTGCGATAGTAGAACGAGAA |
| Q-*arnA*-R | ACCCATCCGGACACGGCTGGAGAAA |
| Q-*lpxA*-F | ACGAAGGTGGGCAGCGATAAC |
| Q-*lpxA*-R | AATTGCGAAGTCATCAACCGATACG |
| Q-*lpxB*-F | AAGTGGTTGTTCCGCTGGTAAATG |
| Q-*lpxB*-R | TCGCTCGCATACATCGCTTCC |
| Q-*lpxC*-F | AGTGCTGCACCGTTCGTCTATC |
| Q-*lpxC*-R | TTCAGCCCACTTGTCGCCATC |
| Q-*lpxD*-F | GCATCATCGCCTGGGTGTTAAAG |
| Q-*lpxD*-R | ATTGTCGCCTGTCTGGTCTACG |
| Q-*lpxL*-F | ATGAAAAAGGCGGAGCGAGA |
| Q-*lpxL*-R | AACCCGTCACGGTAAAGCAT |
| Q-*ramA*-F | CGCTCGGGTGTACGACATCTG |
| Q-*ramA*-R | AGTGCGTCTGACTGTGGTTCTC |
| Q-*ramR*-F | GCGGCACAGTTCATGGAGTTC |
| Q-*ramR*-R | CACCATCACGCTTCCCAAAGAG |
| Q-*acrA*-F | GCCTGGTAATTCGGTGGTGATTTG |
| Q-*acrA*-R | CCTCTGGCGGTCGTTCTGATG |
| Q-*acrB*-F | CGATAAGAGCACGCACCACTACAC |
| Q-*acrB*-R | GCTTGGCAGACGGACGAACAG |
| Q-*oqxB*-F | TGCCGATGACGATGCTCTCC |
| Q-*oqxB*-R | CATAATGCCTTTGCCCTGAATTTCC |
| **Primers for sequencing** | |
| *phoP*-F | GTGGCGTAAAATCCCTCTCATT |
| *phoP*-R | GTGGCGTAAAATCCCTCTCATT |
| *phoQ*-F1 | TCGCATTTTTCACATAACGGGTTAA |
| *phoQ*-R1 | GTGAGCTGGAAGAACATCATCG |
| *phoQ*-F2 | CTTTTCAGCAGGCGGTTGAGG |
| *phoQ*-R2 | TACCTGTTCGAATTACGCTAAATGAG |
| *mgrB*-F  *mgrB*-R | CACCTTGAGAAAAAATGCGTATTAC  GGTTTTTAGACGGAGTGTGGAG |
| *acrA*-F | GAGGTTTACATATGAACAAAAACAGAG |
| *acrA*-R | TCCTGTTTAAGTTAAGACTTGGTTTG |
| *acrB*-F | TTAAGACATGCCTAATTTCTTTATCG |
| *acrB*-R | AACAGGTACATCACCAGGAATAC |
| *marA*-F | GTTACAGGCGATGATTAACTACAG |
| *marA*-R | GAGGTATGACGATGTCCAGAC |
| *soxS*-F | GGCAACCTTATGTCGCATCAG |
| *soxS*-R | CTGAGGGCGATTAATTCAACTG |
| *oqxB*-F | GAAAACGGTTGGATCGTAAGGT |
| *oqxB*-R | CCTGAGAATCCTATCCATGGAC |
| *ramA*-F | GCAGCATGACCATTTCCGCTC |
| *ramA*-R | GAAAACAGCTCAGTGCGTCTG |
| *lpxA*-F | TAGCCGGGAGTCCTGATACG |
| *lpxA*-R | CGACTGTCGACCATTAACGAAT |
| *lpxB*-F | AGGGGTCTGATTCGTTAATGGT |
| *lpxB*-R | GATAAACAAATTCCATCATTTTGCTAA |
| *lpxC*-F | TTGGCGAGATTATACGATGATCAAA |
| *lpxC*-R | CGAAACGGTTTGAACCGTTACG |
| *lpxD*-F | GTTAAATAAGTAATGCCTTCAATTCG |
| *lpxD*-R | CAACGGATGACGCTTAATCTTG |
| *lpxL*-F | CTGCCTCAGTGCTGGACG |
| *lpxL*-R | GACACGCTAAAGGATAATTTTCTCAT |

(2) **Used for *Enterobacter kobei.***

| Primers | Sequence (5' → 3') |
| --- | --- |
| Q-*phoQ*-F | TCTCACTGCGGGTTCGTTTT |
| Q-*phoQ*-R | TCGCCACGTAACAGTCGAAA |
| Q-*arnA*-F | CACGCCTATCGAGTACACCC |
| Q-*arnA*-R | GTCGTATTTCACGCAGTCGC |
| Q-*lpxA*-F | CAGTACAGGGTGGTGGGTTGAC |
| Q-*lpxA*-R | CTGCCAGCGTTGCGTTGTTG |
| Q-*lpxB*-F | TGGTGGTCGGTTATCGTATGAAGC |
| Q-*lpxB*-R | GAGGCTGGCACTCATCCTGTAAG |
| Q-*lpxC*-F | CGCATCAAAGAGACCGTTCGTG |
| Q-*lpxC*-R | CAGCAGAGAAGTTCATCGCATAGC |
| Q-*lpxD*-F | CCGTGAACAACTGGCTCAATGC |
| Q-*lpxD*-R | TGCGGCGTGGTATCAAGAATTTG |
| Q-*lpxL*-F | GGCCGCATGAGGATCAATAGC |
| Q-*lpxL*-R | ACAGGAAAGACATGACCCAGTT |
| Q-*ramA*-F | GCGGGATCTGCGTGAATCGG |
| Q-*ramA*-R | GCGTCTGACTGTGGTTTTCCTTG |
| Q-*ramR*-F | TTCACCGCCATTTCCGTTTCG |
| Q-*ramR*-R | AGAACATACCCGCAATATCTGGAAC |
| Q-*acrA*-F | GCACCAATCCAACCGCACTTC |
| Q-*acrA*-R | CACCAGCCATTTATCGCCAATCG |
| Q-*acrB*-F | TGGCAGACGAACGAACAGATAGG |
| Q-*acrB*-R | TGACAAGAGCACGCACCACTAC |
| Q-*oqxB*-F | AACCTATCTGGGCTCGTCTTATGTG |
| Q-*oqxB*-R | TTCGCAATATCCTCCACGCTCTC |
| *phoP*-F | CATATTCTGGGAGAAAAGATGATGC |
| *phoP*-R | ATGGCGCAAAATCTGTCTCATTTAG |
| *phoQ*-F1 | TCGCATTTTTCACATAACGGGTTAA |
| *phoQ-R1* | GCGAGCTTGAGGAACACCATC |
| *phoQ*-F2 | CTTTTCAGCAGGCGATTGAGAT |
| *phoQ-R2* | TACCTGTTTGAATTACGCTAAATGAG |
| *mgrB-F*  *mgrB-R* | CACCTTAAGAAAAAATGCGTGCTAC |
|  | GGTCTTTAGACGGAGTGTGGA |
| *acrA*-F | GGCTCCTGTTTAAGTTAAGACTTG |
| *acrA*-R | TCGAGGTTTACATATGAACAAAAACAG |
| *acrB*-F | CGGTATCAGTGCGGTTCTACC |
| *acrB*-R | CTCTCTGAAAGACTGGGCGG |
| *marA*-F | GTTACAGGCGATGATTAACTACAG |
| *marA*-R | GAGGTATGACGATGTCCAGAC |
| *soxS*-F | TGCACTAAGGGTGATTAATTCAACTG |
| *soxS*-R | GGGCAACCTTATGTCGCATCA |
| *oqxB*-F | TTTTGTTTTCTGCAACATCCCTATG |
| *oqxB*-R | TTGGCTATGGCGCATTGCAGA |
| *ramA*-F | GAAGGGGAGAGCAGTATGACC |
| *ramA*-R | GTGTGTTGTCATTTTCAGTGCG |
| *lpxA*-F | GCCGGGAGTCCTGATACGT |
| *lpxA*-R | GACGACTGTCGACCATTAACG |
| *lpxB*-F | TGATTCGTTAATGGTCGACAGTC |
| *lpxB*-R | TCCAGCACTGCATCGGCC |
| *lpxC*-F | GAGATTATACGATGATCAAACAAAGG |
| *lpxC*-R | CGGTGTGAACCGTTAAGCCAG |
| *lpxD*-F | TAAATAAGTAATGCCTTCAATTCGAC |
| *lpxD*-R | TTAGTCTTGTTGATCGATCTTACGC |
| *lpxL*-F | GGCCGCATGAGGATCAATAGC |
| *lpxL*-R | ACAGGAAAGACATGACCCAGTT |
